# Supplementary material for: Gene Expression Profiling of MicroRNAs in HPV-Induced Warts and Normal Skin
Source: Biomolecules. 2019 Nov 21;9(12):757. doi: 10.3390/biom9120757 (PMC6995532; doi:10.3390/biom9120757)
Supplement: Supplementary file 1 [file biomolecules-09-00757-s001.pdf]

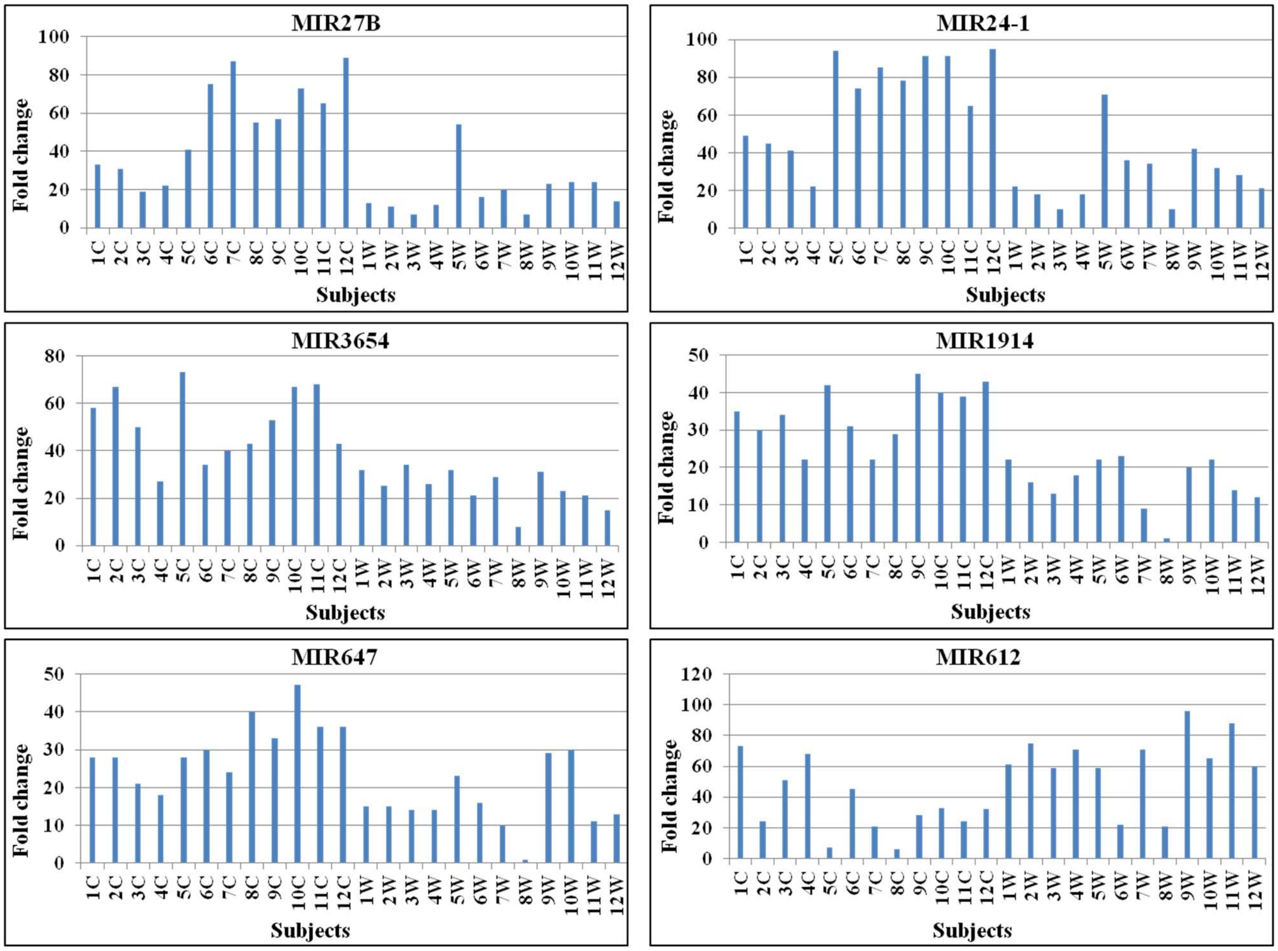

Figure S1: Fold change for control subjects (1C-12C) and wart subjects (1W-12W) plotted for the six differentially expressed microRNAs.

Table S1: Predicted disease associations of the candidate miRNAs

miRTarBase ID	Disease	PubMed ID (PMID)
hsa-mir-24-1	B-cell chronic lymphocytic leukemia	16103053
	Breast cancer	16192569 ǀ 18376396
	Cancer	18429962 ǀ 17111382 ǀ 19891660 ǀ 17922033 ǀ 18923441
	Cardiomyopathy, dilated	17606841
	Cardiovascular disease	17712037 ǀ 17108080
	Chronic lymphocytic leukemia (CLL)	16103053
	Colorectal cancer	16103053 ǀ 16505370 ǀ 16461460
	Dermatitis, atopic	17622355
	Gastric cancer	16103053 ǀ 16461460
	Gastrointestinal cancer	19030927
	Glioblastoma	16103053
	Glioblastoma multiforme (GBM)	16039986
	Hematological disease	16192569
	Hodgkin's lymphoma	19177201
	Leukemia, acute myeloid	18308931
	Leukemia, chronic lymphatic	16251535
	Lung cancer	16192569 ǀ 18375788 ǀ 18766170
	Melanoma and neural system tumor syndrome	16754881 ǀ 18379589
	Miyoshi myopathy (MM)	17942673
	Muscular disorder	18182067 ǀ 17243163
	Myopathy, nemaline, 3	17942673
	Neuroblastoma (NB)	17283129
	Pancreatic cancer	16103053 ǀ 16192569 ǀ 17149698 ǀ 16966691 ǀ 16461460
	papillary thyroid carcinoma (PTC)	16103053
	Parkinson's disease	17761882
	Pituitary Adenomas	17260024
	Prostate cancer	16192569 ǀ 18459106 ǀ 16461460
	Schizophrenia	17326821
	Squamous cell carcinoma, head and neck	16192569
	Stroke	19888324
	Supravalvar aortic stenosis	17712037
	Systemic lupus erythematosus (SLE)	18042587
	Thrombocytopenic purpurea, autoimmune	18042587

hsa-mir-27b	Acute lymphoblastic leukemia (ALL)	18379589
	Acute myeloid leukemia (AML)	18379589
	Alzheimer Disease	18525125
	Breast cancer	16192569
	Cancer	18923441 | 19891660
	Cardiac hypertrophy	17344217 | 19074899
	Colorectal cancer	17330104 | 16609010 | 17363563 | 16505370 | 18607389
	Esophageal cancer	19737949 | 18242245
	Gastroesphageal reflux	19737949
	Gastrointestinal cancer	19030927
	Glioblastoma multiforme (GBM)	17363563
	Hematological disease	16192569
	Leukemia, acute myeloid	17363563
	Leukemia, chronic lymphatic	15284443
	Leukemia, chronic myeloid	17363563
	Leukemia/lymphoma, chronic B-cell	17363563
	Lung cancer	19567675 | 16192569 | 18766170 | 16530703
	Melanoma and neural system tumor syndrome	16754881
	Multiple myeloma (MM)	17363563
	Muscular disorder	18182067
	Myopathy, nemaline, 3	17942673
	Neuroblastoma (NB)	17283129
	Non-alcoholic fatty liver disease (NAFLD)	18379589
	Oral Squamous Cell Carcinoma (OSCC)	18456660
	Pancreatic cancer	16192569 | 16966691
	Prostate cancer	18456660 | 18812439 | 17616669 | 16192569 | 18459106
	Squamous cell carcinoma, head and neck	18381414 | 16192569
	Stroke	19888324
	Supravalvar aortic stenosis	17712037
hsa-mir-612	Lupus Nephritis	19435529
	Systemic lupus erythematosus (SLE)	18998140
